# The anti-infective potential of human milk oligosaccharides in carbohydrate powder in implant-associated infection with Staphylococcus aureus

**DOI:** 10.1371/journal.pone.0342316

**Published:** 2026-03-23

**Authors:** Julian Koettnitz, Franz Koettnitz, Tobias Tiemann, Gael Vos, Kevin Pagel, Silke Zechel-Gran, Eugen Domann

**Affiliations:** 1 Ruhr-University Bochum (RUB), Medical Faculty, University Hospital Auguste-Viktoria, Department of General Orthopedics, Bad Oeynhausen, Germany; 2 Ammeva GmbH, Werder (Havel), Germany; 3 Institute of Chemistry and Biochemistry, Freie Universität Berlin, Berlin, Germany; 4 Institute of Hygiene and Environmental Medicine c/o Institute of Medical Microbiology, DZIF – German Centre for Infection Research Biomedical Research Facility Seltersberg (BFS), Giessen, Germany; Tribhuvan University, NEPAL

## Abstract

**Introduction:**

Periprosthetic joint and implant-associated infections remain serious complications despite highly standardized diagnostic and therapeutic protocols. Repeated revision procedures substantially increase morbidity and the risk of therapeutically uncontrollable infection scenarios. This pilot study, we evaluated the effect of HMO (human milk oligosaccharides)-containing carbohydrates on S. aureus biofilm formation using crystal violet assays and implant models.

**Methods:**

S. aureus EDCC 5055 biofilm formation was quantified in the presence of HMO-containing carbohydrates (HMO-C) at concentrations of 5%, 7%, 9%, and 11% and lactose-alone in 96-well plates. After processing, the plate contents were read out using a 595nm Phomo plate reader (Anthos Mikrosystems, Krefeld, Germany). Biofilm activity was further evaluated on titanium disks pre-incubated with HMO-C solutions. Bacterial growth kinetics were also analyzed in TSB with the 5% to 11% HMO-C solutions.

**Results:**

The results demonstrated a significant reduction in S. aureus biofilm formation with the addition of HMO-C (HMO-C-5%: mean value (x¯)=0.271nm; p = 0.021; HMO-C groups 7%−11%: x¯ = 0.211nm, 0.179nm, 0.147nm; All p = 0.001) against the positive control EDCC 5055 (PC) (x¯ = 0.335nm). Lactose (L) alone did not significantly affect biofilm formation (L5%−11%: p = 1.0). No significant biofilm reduction was observed for titanium implants, though medium changes indicated bacterial inhibition at higher HMO-C solutions (9%−11%: x¯ = 0.844nm, 0.940nm; Both p = 0.001).

**Conclusion:**

Media supplemented with HMOS-C significantly reduced S. aureus ED CC 5055 biofilm formation in vitro in the crystal violet microtiter plate assay. Bacterial invasion on titanium could not be demonstrably changed, but S. aureus growth curve was significantly reduced. Further studies with optimized implant models and standardized HMO formulations are warranted to clarify the translational potential of HMO-C for the prevention of implant-associated infections.

## Introduction

Human milk oligosaccharides have emerged as a promising avenue in the fight against bacterial infections, particularly in the context of biofilm formation and implant-associated infections. These complex carbonhydrates, which are the third most abundant solid component in human milk after lactose and lipids, have garnered significant attention for their multifaceted roles in infant health and potential therapeutic applications [1–3]. HMOs are unique to milk and are present in concentrations ranging from 5 to 23 mg/L, far exceeding the oligosaccharide content found in bovine milk. The complex carbohydrates are described as prebiotic, immunomodulatory and anti-inflammatory and have been shown to shape the gut microbiota [1,4,5]. Recent research has identified their potential as antimicrobial and antibiofilm agents, particularly against pathogenic bacteria such as Staphylococcus or Streptococcus species. These bacteria can form biofilms on medical devices and implants, creating a protective environment that enhances their resistance to antibiotics and host immune responses [6,7]. The ability of HMOs to disrupt biofilm formation and inhibit bacterial growth offers a novel approach to combating these challenging infections. Studies have demonstrated that HMOs exhibit antibiofilm activity against various strains of *Staphylococcus aureus* (*S. aureus)*, including MRSA [8,9].

The mechanism by which HMOs exert their antibiofilm effects is multifaceted. They can interfere with bacterial adhesion to surfaces, disrupt the extracellular polymeric substances (EPS) that form the biofilm matrix, and modulate bacterial gene expression related to biofilm formation [9,10]. However, human milk oligosaccharides are not the only substances currently being investigated for their anti-infective properties. Other studies have demonstrated the promising antibiofilm properties of insect and plant-based oligosaccharides. For example, chitosan oligosaccharides (COS) have been utilized to functionalize implant surfaces, as shown in a research where 3D-printed PLA constructs were coated with a combination of zinc, halloysite nanotubes, silver, and COS. This approach significantly reduced biofilm formation by *Staphylococcus aureus (S. aureus)* and *Staphylococcus epidermidis (S. epidermidis)*, both common pathogens in implant-related infections [11]. In another study, COS conjugated with streptomycin, resulting in an enhanced ability to disrupt biofilms of *Pseudomonas aeruginosa (P. aeruginosa)*, highlighting the synergistic effects of oligosaccharide-antibiotic combinations. In another study, COS was conjugated with streptomycin, resulting in an enhanced ability to disrupt biofilms of *Pseudomonas aeruginosa*, highlighting the synergistic effects of oligosaccharide-antibiotic combinations [12]. Furthermore, alginate oligosaccharides (AOS) have also been investigated for their antibiofilm activity, with studies demonstrating their efficacy against *S. aureus* and *Streptococcus agalactiae (S. agalacticae)* biofilms, especially when used in combination with antibiotics [13].

In summary, oligosaccharides represent a new class of potential bacterial biofilm defense and infection reduction.

Unlike many traditional antimicrobials that rely on single mechanisms of action, HMOs act through multiple pathways, thereby making it substantially less likely for bacteria to develop resistance.

Therefore, HMOs could represent a novel, nature-inspired strategy for preventing and treating implant-associated infections, with their ability to inhibit bacterial adhesion, disrupt biofilms, modulate pathogen behavior, and enhance host defenses.

The primary objective of this study was to systematically evaluate the anti-biofilm and antibacterial effects of HMO-enriched carbohydrate powder against *S. aureus* through quantification of the impact of different HMO-C concentrations using the crystal violet assay, assessing the ability of HMO-C solutions to inhibit biofilm development and bacterial colonization on titanium implant surfaces and investigating the influence of HMOs on bacterial growth kinetics. The findings of this study may contribute to the design of novel, nature-inspired strategies to reduce the burden of biofilm-related complications in orthopedic and trauma surgery and to address the growing problem of antibiotic resistance.

## Materials and methods

### Bacterial strain

The Isolate *S. aureus* EDCC 5055 is confirmed by PCR methods and MALDI-TOF as *S. aureus* and is stored in Eugen Domann Culture Collection (EDCC), number EDCC 5055 (culture No. DSM 28763/ BacDive ID: 24582). *S. aureus* EDCC 5055 is a clinical wound isolate strain that was originally isolated from a human wound infection (Germany/Giessen). The strain is extensively used as a model organism for research into implant-associated infections in animal models [14,15]. EDCC 5055 is characterized by strong hemolysis and pronounced biofilm formation and is therefore particularly suitable for studies on biofilm and infection research on implants. The complete genome (2,794,437 bp) and a plasmid (27,437 bp) have been sequenced and are publicly available. The strain is known for its high biofilm formation and is frequently used in in vitro and in vivo models for investigating implant infections and antibacterial strategies.

### Lactose

The industrial produced Edelweiß-lactose is produced by the company Peter Kölln KGAA (ProcutNo.: 01298935, Peter Kölln GmbH & Co. KGaA GER) and is available for purchase everywhere. The powder is 99,5% lactose-monohydrate and the chemical structure is β-galactose + α-glucose (β-1,4) with an α-/β-lactose-ratio of 2:3. The product is made by cow’s milk. There are no structural differences of the composition between cow’s and human’s lactose [16].

### Implant material

The implants of the company Aesculap AG were provided in the sizes 3x2x0.3cm in the material Titan (No catalog number available).

### HMO-containing carbonhydrates

The industrial produced and sterile HMO-C (No catalog number available) was provided by the company Ammeva GmbH in cooperation with the Free University Berlin. The HMO-C were delivered as a powder in the natural composition with approximately 80% lactose and 20% human milk oligosaccharides and in a human milk oligosaccharide saturated composition with an almost 50–50% composition.

### Preparation of the Carbohydrate solutions (HMO-C)

The carbohydrate solutions were prepared in concentrations of 5%, 7%, 9% and 11%. For this purpose, the carbohydrate powder was weighed out into a 50 ml Greiner tube using a spatula cleaned in alcohol. Subsequently, 6 milliliters of TSB (tryptic-soc-broth) medium (No: STBMTSB12, Merck KGaA Darmstadt GER) were added under the sterile bench and vortexed. The medium was then incubated in an incubator at 37 Celsius degrees for 30 minutes and then vortexed again until all parts were visually dissolved. For 6 ml TSB-media, concentrations of carbohydrates of 0,3g/6 ml, 0,42g/6 ml, 0,54g/6 ml and 0,66g/6 ml were used to achieve the above-mentioned concentrations.

### Preparation of the overnight culture

For the overnight culture, the standard protocol of the institute was used. Brain heart infusion (BHI) was filled into 250 ml Erlenmeyer flasks. Next, the bacteria were scraped off the blood agar plate with a sterile Drigalski spatula and carefully stirred into the prepared Erlenmeyer flask. Overnight cultures (OOC) were prepared in BHI medium in a shaking incubator at 180 rpm (rotations per minute) at 37°C. The next morning, the OOCs were diluted 1:20 in fresh BHI (Brain-Heart Infusion).

### Crystal-violet-Biofilm-assay

The biofilm assay was performed once for each HMO-C solution (80/20 and 50/50), but multiple technical replicates (eight replicates per row) were done. A 96-well plate was used for biofilm development, with all HMO-C solutions (5%,7%,9% and 11%) with EDCC 5055, a positive control, and a negative control tested. The biofilm-forming S. aureus EDCC 5055 strain served as the positive control, and TSB medium was used as the negative control.

An overnight culture of S. aureus EDCC 5055 was prepared in 20 ml TSB at 37°C in an incubator with shaking at 150 rpm (IKA Werke, Staufen, Germany). For the assay, 2.5 ml of each HMO-C solution (concentrations 5%, 7%, 9%, and 11%) was taken from the 50 ml Greiner tube. The solution was non-sterile filtered into a fresh 5 ml Eppi-Cap. Then, 3.5 ml was drawn up with a sterile, 0.22 µm filter syringe and pressed into a sterile 5 ml cap. The filtered HMO-C solution was then pipetted (3 ml) into a fresh 5 ml cap. After overnight incubation of the S. aureus culture (ONC), 1:100 dilutions of the test solutions with S. aureus were prepared. For this, 2.5 ml non-sterile filtered HMO-C solution was mixed with 25 µl ONC, 3 ml sterile filtered HMO-C solution was mixed with 30 µl ONC, and 5 ml TSB was mixed with 50 µl ONC.

On the following day, the overnight cultures were diluted 1:100 in TSB, vortexed, and 200 µl per bacterial strain, positive control, and negative control were pipetted into each well of an eight-well row (e.g., A–H). The plates were incubated for 24 hours in a sealed CO2 incubator.

After incubation, the medium was carefully removed from the wells without disturbing the biofilm. Each well was washed twice with 200 µl sterile water. After washing, 220 µl of 0.01% crystal violet (No.: C6158-100G, Sigma-Aldrich, Merck KGaA, Darmstadt, Germany) was added to each well. After 15 minutes of incubation in the dark, the dye was removed. The wells were washed again, and the colored crystals were dissolved in acetic acid for 10–20 minutes. Then, 125 µl of the dissolved solution per well was transferred in the same order into a flat-bottomed 96-well plate and measured at 595 nm in the plate photometer. The reduced quantity of 125µl is added to the wells to prevent contamination of the device.

### Titan-plate-experiment

The HMO-C concentrations used were 0, 5, 7, 9, and 11% in 4 ml TSB medium. Prior to use, 200 µl of each dilution (0–11%) was plated onto blood agar plates to verify sterility. No bacterial growth was observed after overnight incubation at 37°C in a CO₂ incubator. Four different HMO-C concentrations and appropriate positive and negative controls were tested. The assay utilized the biofilm-forming S. aureus EDCC 5055 strain as the positive control.

In the first step, 3960 µl of medium (comparison control) or medium with HMO-C was added to the pre-incubation tubes and incubated for two days. S. aureus was then added and further incubated to allow biofilm formation. For preparation, HMO-C powder was weighed into 50 ml Greiner tubes to achieve final concentrations of 5%, 7%, 9%, and 11% in TSB, using a sterile spatula. The milk powder was weighed into 15 ml Greiner tubes for incubation material. To improve solubility, the tubes were incubated at 37°C for 20 minutes and then vortexed. Sterile filtration (0.22 µm) was performed, and the filtered solution was transferred to sterile 5 ml caps. One piece of titanium was placed in each of five 15 ml Greiner tubes. Four milliliters of TSB without titanium served as the medium negative control, which was incubated overnight at 37°C. Subsequently, 3960 µl of each HMO-C–TSB solution (0, 5, 7, 9, 11%) was added to the remaining five 15 ml Greiner tubes containing titanium. All tubes underwent a 24-hour pre-incubation at 37°C in a CO₂ incubator (all tubes/media were sterile at this stage). Then, 40 µl of an overnight S. aureus culture was added to the five tubes with the 3960 µl HMO-C–TSB. The negative control tube with 4 ml TSB remained free of bacteria. Incubation continued for 96 hours at 37°C and 5% CO₂. After the incubation period, the medium was removed under sterile conditions, and the titanium pieces were washed with 10 ml NaCl. Each titanium piece was then placed in a fresh sterile tube. The washing process was repeated. Subsequently, each titanium piece was transferred to a new sterile tube, and 15 ml Greiner tubes were ultrasonicated to detach the biofilm. The resulting suspension was collected, and the tubes were placed in an ultrasonic water bath. The material was mixed after sonication.

Biofilm formation was quantified by plating serial dilutions of the suspension onto BHI agar plates (No.: 1038700500, Merck KGaA, Darmstadt, Germany). Plates were incubated overnight at 37°C, and colony-forming units (CFUs) were counted. The CFU was counted by the Scan 500 (Interscience, Wiesbaden, Germany), which is a fully automated, image-based system for counting colonies on Petri dishes. The BHI agar plates were placed inside the device, which automatically identifies and marks all colonies in standardized and repeatable reproducibility.

### Bacterial growth curve

The day before the experiment, prepare an overnight culture by inoculating 20 ml TSB with Staphylococcus aureus EDCC 5055 from a blood agar plate and incubate at 37°C with shaking at 150 rpm. For the preparation of biofilm solutions, transfer 2.5 ml from each HMO-C concentration (5%, 7%, 9%, 11%) in the 50 ml Greiner tube into a fresh 5 ml Eppendorf tube. Using a syringe and cannula, draw up 3.5 ml per concentration and filter through a 0.22 µm sterile filter into a 5 ml cap. Pipette 3 ml of the sterile HMO-C solution into a fresh 5 ml cap.To prepare the bacterial suspension, make 1:100 dilutions of S. aureus in the test solutions. For the assay, pipette 200 µl of each test solution and control solution into three wells each of a 96-well Tecan microtiter plate. Cover the plate and place it directly into the Tecan device (Infinite 200Pro). Start the measurement at 23:59 (highest value) and record under the following conditions: wavelength 600 nm, temperature 37°C, kinetic interval 20 minutes, total duration 360 seconds.

### Statistical analyses

All statistical analyses were performed using the software IBM SPSS version 29 (IBM, Armonk, NY, USA). Metric-scaled data were analyzed by mean, standard deviation, and variance. For the analysis of metric and nominally scaled variables, the T-test for independent samples (including Welch an Levene-Test) and the analyses of variance (ANOVA) were used. The effect sizes Cohen’s d (small 0.20; medium 0.50; large 0.80) and 95% interval were used. For nominal scales with more than two values the univariate linear regression analyses with the Post-Hoc-Bonferroni-Test was used. The significance level was set two-sided with α = 0.05. The Bonferroni post hoc test was used for groups n= ≥ 3 and multiple testing to control the p values for their actual quality and relevance. Furthermore, for multiple testing the alpha level was divided through the number of tests, which set the threshold to 0.003.

## Results

### Crystal-violet-Biofilm-assay

**Fig 1:**
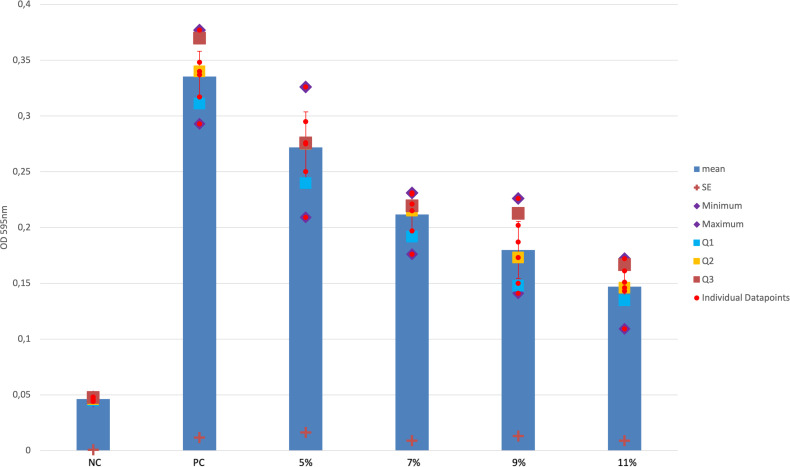
Biofilm formation of S. aureus EDCC 5055 and EDCC 5055 with 5 to 11% HMO-C in 80/20 saturation of lactose to HMOs. NC = negative control, PC = positive control, SE = standard error, Q1 = 1. quartile (25%), 2. quartile (50%), 3. quartile (75%), red line (two-sided error indicator with closure) = 95%Confidence Intervall..

**Fig 2:**
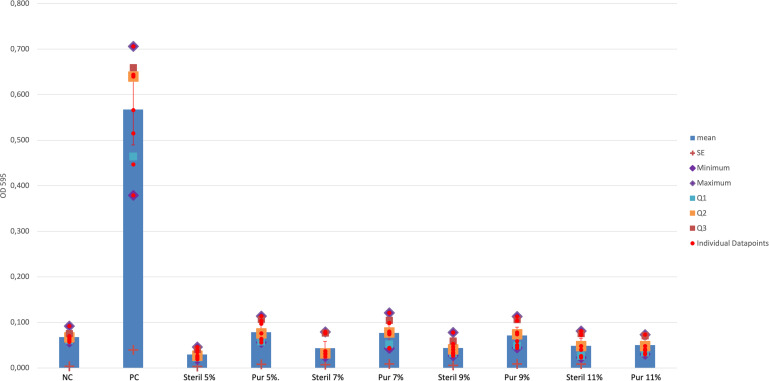
Biofilm formation of PC-S. aureus EDCC 5055 and EDCC 5055 with a 100µl TSB-media containing concentrations of 5 to 11% HMO-C in 50/50 saturation of lactose and HMOs; Furthermore, the columns present “sterile” and “pure”. In the sterile group, the 100 µl TSB with the respective HMO-C concentration 5-11% was added to the wells through a filter. s = sterile, p = pure. HMO-C = HMO-containing carbohydrates, NC = negative control, PC = positive control, SE = standard error, Q1 = 1. quartile (25%), 2. quartile (50%), 3. quartile (75%), red line (two-sided error indicator with closure) = 95%Confidence Intervall.

The Crystal-violet-Biofilm-assay revealed no significant difference in the development of the biofilm of *S. aureus* EDCC 5055 between the positive control (PC) and the 80/20 saturated HMO-C group in 5% concentration in the media (PC to HMO-C-5%group: p = 0.021 (*Bonferroni corrected), 95%CI = 0.0072/0.11998;), but for all other 80/20 saturated HMO-C-groups 7–11% concentration in the media (PC-all other HMO-C-groups: p = 0.001 95%CI = 0.698/0.899) as well as the 50/50 saturated HMO-C groups (p = 0.001 (*Bonferroni corrected), partial eta square (η^2^_p_)=0.820). For the 80/20 saturated HMO-C groups a significant difference in the biofilm reduction was found between the 5% and 9–11% solutions (p = 0.001 (*Bonferroni corrected), 95%CI: (9%)=0.03966/0.14434, (11%)=0.7249/0.17717). The Figs 1 and 2 show the strength of biofilm for the 0% to 11% HMO-C groups with *S. aureus* EDCC 5055 in the two different concentrations of lactose to HMOs.

**Fig 3:**
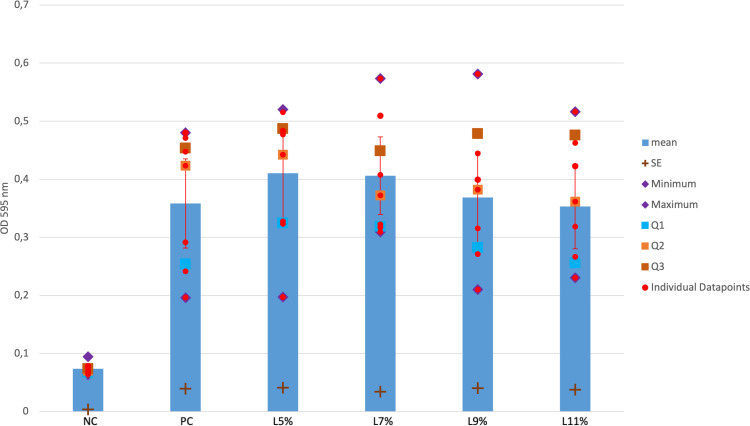
Biofilm formation of PC-S. aureus EDCC 5055 and EDCC 5055 with 5 to 11% containing concentrations of lactose. NC = negative control, PC = positive control, L = lactose SE = standard error, Q1 = 1. quartile (25%), 2. quartile (50%), 3. quartile (75%), red line (two-sided error indicator with closure) = 95%Confidence Intervall.

To assess the anti-biofilm efficacy of lactose, the crystal violet test was repeated using lactose alone. No significant biofilm reductions were observed with lactose compared to PC (PC-5% and 7%: p = 0.951, 0.966 (*Bonferroni corrected); η^2^_p_=0.610; PC-9% and 11%: p = 1.0(*Bonferroni corrected); η^2^_p_=0.610) (see Fig 3).

### Titan-plate-experiment

**Fig 4:**
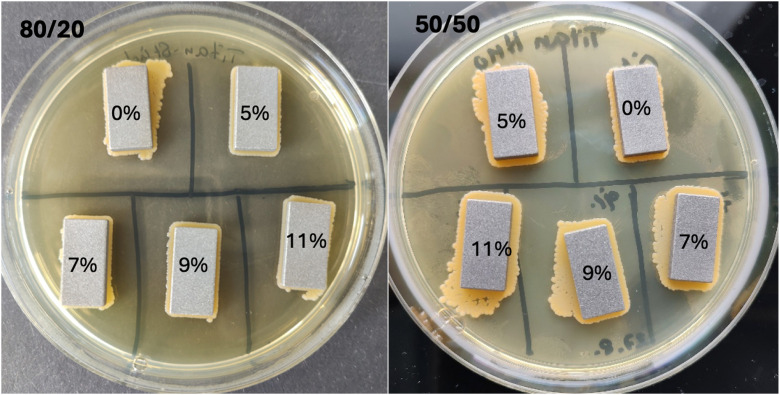
Titan plates with the different HMO-C-concentrations - on the left the 80/20 lactose to HMO-C-group to the right, the 50/50 lactose to HMO-C group; The percentage shows the concentration of HMO-C in the media from 0-11%. The pictures were taken for visibility of the titan plate samples und the BHI-broth plates.

The titan-plate-experiment with the concentrated *S. aureus* EDCC 5055 media solution was diluted into 1:2. 100 µl was plated on each plate and three plates per group were used. The EDCC 5055 group and the HMO-C groups did not reveal any significant biofilm reductions. The Colony-forming-units were not significantly different. The CFU values for the Positive control and the 5%, 7%, 9% and 11% group of the 80/20 percent carbohydrate group (lactose to HMOs) were 916, 807, 969, 801 and 800 CFU per 100µl. The CFU values for the Positive control and the 5%, 7%, 9% and 11% group of the 50/50 percent carbohydrate groups (lactose to HMOs) were 1543, 1898, 2080, 1227 and 1609 CFU per 100µl. Fig 4 shows the titan plates with the 80/20 HMO-C groups and the 50/50 HMO-C groups.

CFU was measured after plating on BHI nutrient plates. No significant difference could be measured between the HMO-C-concentrations 5–11% for each (p = 1.0, Cohens d = 0.1) and the Positive control. Only cell layers were detected in the plating of 200 µl in all groups.

### Bacterial growth curve (only 50/50-HMO-C-groups)

**Fig 5:**
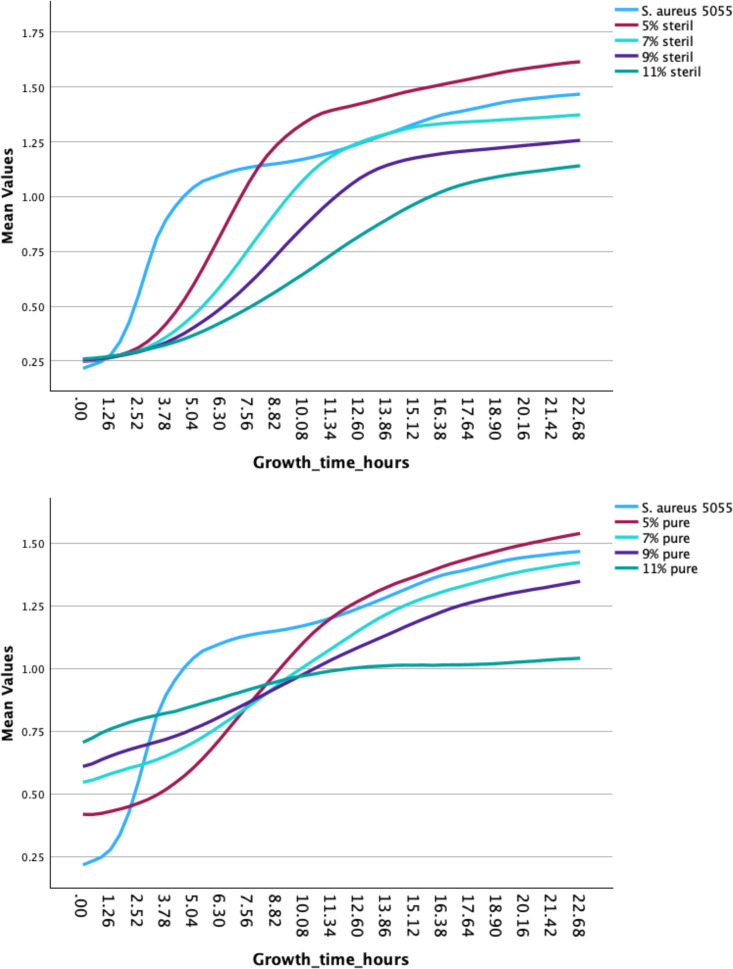
Bacterial Growth curve with S. aureus EDCC 5055 and different 50/50HMO-C-concentrations 5%, 7%, 9% and 11% sterile and pure filtered. On the left and right side S. aureus EDCC 5055 shows a typical growth behavior, from lag phase to exponential and then to the stationary phase. The 5% HMO-C-concentration sterile and pure showed an enhanced exponential growth above the S. aureus positive control. The 7% group revealed an almost similar growth. In the 11% group the exponential phase is clearly flattened and the stationary phase was reached earlier. The increased density at the start of the measurement of the non-sterile filtered HMO-C can be explained by the increased turbidity of the solution without filtration.

The bacterial growth curve showed significant growth inhibition of *S. aureus* EDCC 5055 in the 9% and 11% sterile 50/50-HMO-C-groups (p = 0.002 and 0.001 (*Bonferroni corrected); 95%CI = 0.06766/0.499039 and 0.194681/0.626054), but not in the 5% and 7% sterile HMO-C-groups (p = 1.0 and 0.287 (*Bonferroni corrected)). In the pure group, only the 11% showed a significant growth inhibition of *S. aureus* EDCC 5055 (p = 0.001 (*Bonferroni corrected); 95%CI = −0.28197623/ −0.8377759), but not in the other groups 5–9% pure HMO-C-groups (p = 1.0, 0.990, 0.431). Fig 5 shows the curve progression of the individual samples.

## Discussion

The present investigation demonstrates the anti-infective potential of human milk oligosaccharides (HMOs) in carbohydrate powder (HMO-C) against implant-associated *Staphylococcus aureus* infections, with particular emphasis on biofilm formation and bacterial growth inhibition. Although our findings reveal significant biofilm reduction in vitro using crystal violet assays, the translation of these effects to the implant surface titanium remains limited. This discussion addresses the implications and limitations of our results.

The crystal-violet biofilm assay revealed significant reductions in *S. aureus* EDCC 5055 biofilm formation when exposed to HMO-C solutions. Both the 80/20 and 50/50 saturated HMO-C-powder groups demonstrated statistically significant differences (PC to HMO-C-5%group: p = 0.021, 95%CI = 0.0072/0.11998; PC-all other HMO-C-groups: p = 0.001 95%CI = 0.698/0.899) compared to the positive control. This aligns with previous studies that have shown HMOs to exhibit antibiofilm activity against various strains of *S. aureus*, including methicillin-resistant *S. aureus* (MRSA) [8,17,18]. Another study examined the anti-biofilm activity of 3′-sialyllactose (SL) and 6′-SL against *Clostridium difficile*. Both groups showed a reduced ability to adhere, as well as a reduction in the expression of the cell wall protein cwp84 [17]. Another study showed the repression of biofilm formation in *Acinetobacter baumanii* at al. concentration of only 2.5 mg/ml, but no growth inhibition was shown [19].

The observed dose-dependent effect in this study, particularly in the 50/50 saturated CHP groups, suggests that higher concentrations of HMOs may be more effective in preventing biofilm formation. This concentration-dependent activity is consistent with findings from other studies, indicating that HMOs can interfere with bacterial adhesion and disrupt the extracellular polymeric substances (EPS) that form the biofilm matrix [1,20].

The *S. aureus* EDCC 5055 strain was selected as the model organism for this investigation because it is a clinical wound isolate extensively utilized in implant-associated infection research due to its potent hemolytic activity and robust biofilm-forming capacity. Therefore, EDCC 5055 provides stringent and clinically relevant models for evaluating anti-biofilm interventions on orthopedic implants [14,21,22]. However, the exclusive use of a single bacterial strain limits the generalizability of the findings. Testing other strains, such as ATCC 12228 or ATCC 25923, which are also known for anti-biofilm experiments, would be a logical next step to confirm the effectiveness [23,24].

For isolated lactose, only Kwiecińska-Piróg et al. 2024 described an antibiofilm-effect against E. coli, but only in combination with lactobacilli [25]. In this study, we could not find any anti-biofilm effect of lactose (p = 0.079, p = 0.101, p = 0.635, p = 0.844). Looking at the average values in the graph, it appeared that lactose alone promotes biofilm formation. This confirmed the assessment that the HMOs contained in carbohydrates are responsible for the antibiofilm activity.

Notably, the titanium implant model did not demonstrate significant reductions in biofilm burden or significant differences in colony-forming unit (CFU) counts across the various HMO-C concentrations tested. This discrepancy between the crystal-violet assay and the implant model highlights the complexity of translating in vitro results to more clinically relevant scenarios. The lack of significant effect on titanium surfaces may result from specific interactions between HMOs and titanium surfaces, that differ from the polystyrene plates used in the crystal-violet assay. Recent work on chitosan-based coatings (containing oligosaccharides) illustrates that titanium implants can, in fact, support sustained antimicrobial activity, emphasizing the importance of material-specific HMO delivery systems in orthopedic applications. [26]. Additionally, the dynamic environment around an implant may affect the concentration and efficacy of HMOs. Furthermore, *S. aureus* EDCC 5055 may display distinct adhesion and biofilm-formation behavior on titanium compared with polystyrene.

This discrepancy can also be attributed to the differing mechanical and physiochemical properties of the tested titan and the mechanisms of bacterial adhesion and biofilm development. Biofilm adhesion is governed by complex interactions between the bacterial cell surface and the substrate, including hydrophobic/hydrophilic forces, electrostatic interactions, van der Waals forces, and the presence of a conditioning film of proteins on the implant surface [27,28]. Titanium exhibits unique surface characteristics compared to polystyrene (as used in standard microtiter plate assays), like a micro- or nano-scale roughness to promote tissue integration. The increased roughness can also provide more niches for bacterial attachment and biofilm maturation, making it harder for soluble agents like HMOs to prevent initial adhesion or disrupt established biofilms. Moreover, titan is hydrophilic and forms a stable oxide layer, which can influence the adhesion of both proteins and bacteria [29–31]. Nevertheless, the implant experiment requires repetition using a more efficient and reproducible protocol. Moore et al. 2022 covered and incubated their implants in a 1:1000 dilution and exchanged the media every 24 hours for three days. This could be a starting point for improving the experimental design of this study [32].

Overall, these findings underscore the need for further investigation into the efficacy of HMOs in preventing biofilm formation on various implant materials and under conditions that more closely mimic the in vivo environment.

The bacterial growth curve experiments revealed significant differences in growth patterns between S. aureus EDCC 5055 and the 7–11% sterile HMO groups (p = 0.001). The observed flattening of the exponential phase and earlier onset of the stationary phase in the 11% group suggest that higher concentrations of HMOs may have a growth-inhibitory effect on S. aureus. This growth inhibition aligns with previous research demonstrating the antimicrobial properties of HMOs against various pathogens, including S. aureus [33]. Potential mechanisms include disruption of key metabolic pathways, interference with bacterial communication and population dynamics, or competition with essential nutrients at higher HMO concentrations. In contrast, in 2012 the Hunt et al. working group showed increased growth in a TSB medium in S. aureus and S. epidermidis strains tested, equivalent to the positive control [34]. However, a concentration of 10g/l, i.e., 0.01g/ml, was selected. The concentration was therefore significantly lower than in this study, which makes comparability more difficult. On the other hand, Moore et al. 2021 [35] showed a reduction in the minimum inhibitory concentration (MIC) of the antibiotics in combination with HMOs for the treatment of group B streptococci. Antibiotics typically used in orthopedic surgery, like Vancomycin or Ampicillin showed a halving of the MIC with a doubling of the effectiveness. While HMOs alone showed growth inhibition, emerging evidence suggests enhanced effects when combined with antibiotics. Craft et al. (2018) potentiate the function of aminoglycosides, lincosamides, macrolides, and tetracyclines on a strain specific basis but not β-lactams or glycopeptides that inhibit cell wall synthesis. These findings are notable as GBS has evolved high levels of resistance toward aminoglycosides, macrolides, and tetracyclines [20]. In addition, a study showed that HMOs could significantly improve the immune response in the early stages against *S. aureus* and could stimulate macrophages without leading to an immune response in the absence of *S. aureus* [36]. These findings show the potential of a targeted defense against pathogens. If a combination of antibiotics and HMOs would be able to prevent a further infection and an implant could be remained an additional helpful instrument could prevent patients from extensive removal procedures.

The main limitations of the study lie in the immature experimental design of the implant assay and the lack of in vivo testing with cell lines, which would better assess the biological relevance and therapeutic potential of HMOs in preventing or treating implant-associated infections. Additionally, only one strain, *S. aureus* EDCC 5055 was tested, which currently makes it difficult to draw broad conclusions and should be explained as an important bias of the study. Another weakness is the difference between the incubation time of the crystal violet and the implant experiment (24–96 hours). The different incubation times make a possible comparability of the experiments more difficult. The Biofilm assay was performed only once per HMO-C concentration 80/20 or 50/50. According to biofilm other researches, technical replicates within the same experiment can detect treatment effects, particularly when the effect sizes are substantial, as observed with the significant biofilm reduction [37]. Anyhow, repeating the experiment would strengthen the conclusions by providing biological replicates, increase confidence in the reproducibility of results, accounting for experiment-to-experiment variability, strengthen the statistical power for publication purposes and allow for more comprehensive statistical analysis including ANOVA with proper error terms. The composition of the HMO-containing carbohydrate powder used in our study was not thoroughly characterized. No detailed batch analysis or profiling of individual HMOs was performed. As such, we cannot exclude the possibility, that variation in HMO composition between batches may have influenced the observed effects. This represents a limitation of our study, and in the future a more rigorous standardization and chemical characterization of the HMO-C powder is necessary for future work to control for this potential confounder and to better understand which HMO components are responsible for the anti-biofilm activity. No bacterial growth curve with the 80/20 HMO-C in 5%, 7%, 9%, 11% was done, so that a comparison between the concentrations was not possible to determine whether the higher concentration (50/50) of the powder showed a better reduction in growth.

## Conclusion

As antibiotic resistance continues to challenge orthopedic and trauma surgery, novel, nature-inspired strategies such as HMO-based therapies may play a crucial role in improving patient outcomes and reducing the burden of *S. aureus*-associated infections. The study demonstrates that human milk oligosaccharides (HMOs) in carbohydrate powder exhibit dose-dependent antibiofilm and growth-inhibitory effects against *S. aureus* EDCC 5055, though efficacy varies across experimental models. The Crystal violet assays revealed significant biofilm reduction for the 80/20 satured HMO-C concentrations 7–11% and all 50/50 satured HMO-C concentrations against the positive control (p = 0.001 (*Bonferroni corrected)), while Lactose had no antibiofilm-effect (PC-5% and 7%: p = 0.951, 0.966 (*Bonferroni corrected); PC-9% and 11%: p = 1.0(*Bonferroni corrected)). Titanium implants showed no measurable biofilm reduction despite observable growth inhibition in bacterial cultures (p = 1.0). Growth curve analyses confirmed concentration-dependent suppression of *S. aureus*, with sterile 9 and 11% 50/50-HMO-C solutions (p = 0.002 and 0.001 (*Bonferroni corrected) and the pure 11% 50/50-HMO-C solution (p = 0.001 (*Bonferroni corrected).

It should be expressly mentioned that the limitation of the individual germ selection to only S. aureus EDCC 5055 is a limiting factor for the general validity of this pilot study and only represents one possible additional avenue in orthopedic infection research.

Future work should focus on refining implant models, expanding strain diversity, incorporating cell-based and in vivo studies, and exploring advanced delivery or coating strategies to overcome the mechanical and surface-related barriers identified in this study. Such efforts are essential to fully realize the potential of HMOs as a novel, nature-inspired approach for the prevention and management of implant-associated infections in orthopedic and trauma surgery.
